# Segregation of Trans Mutations in the *CDH23* Gene in an Emirati Family with Sensorineural Hearing Loss

**DOI:** 10.3390/genes15111451

**Published:** 2024-11-10

**Authors:** Mariam Alsebeyi, Abdullah Al Mutery, Mohammad Tehsil Gul, Abdelaziz Tlili

**Affiliations:** 1 Department of Applied Biology, College of Sciences, University of Sharjah, Sharjah P.O. Box 27272, United Arab Emirates; U21102137@sharjah.ac.ae (M.A.); aalmutery@sharjah.ac.ae (A.A.M.);; 2 Human Genetics and Stem Cell Laboratory, Research Institute of Sciences and Engineering, University of Sharjah, Sharjah P.O. Box 27272, United Arab Emirates

**Keywords:** hearing loss, *CDH23* gene, autosomal recessive, next generation sequencing

## Abstract

Background/Objectives: Hearing loss (HL) is a significant global health concern, affecting approximately 1 in every 1000 newborns, with over half of these cases attributed to genetic factors. This study focuses on identifying the genetic basis of autosomal recessive non-syndromic hearing loss (ARNSHL) in a consanguineous Emirati family. Methods: Clinical exome sequencing (CES) was performed on affected members of the family, followed by Sanger sequencing to validate the findings. Specific primers were used for PCR amplification of target *CDH23* exons. Mutations were analyzed using various computational tools to assess their pathogenicity. Results: We identified two heterozygous mutations in the *CDH23* gene: a novel nonsense variant (c.264G>A, p.Trp88Ter) and a missense variant (c.5168G>A, p.Arg1723His). Both mutations were found in trans configuration, suggesting a compound heterozygous state contributing to the phenotype. In silico analysis predicted a significant impact on protein function, potentially leading to the observed ARNSHL. Conclusions: This study emphasizes the complexity of genetic factors in hearing loss, particularly in highly consanguineous populations. The identification of both nonsense and missense mutations in the *CDH23* gene enhances understanding of its role in hearing loss and provides essential insights for genetic counseling and future therapeutic strategies.

## 1. Introduction

Hearing loss (HL) is one of the most prevalent sensory impairments worldwide, affecting nearly 1 in every 1000 births [1]. Environmental and genetic factors can be the causative agents of HL, with hereditary factors accounting for more than half of these cases [2]. Hearing loss can be also caused by a combination of genetics and environmental factors [3]. Genetic hearing loss can be regarded as syndromic (hearing loss accompanied by additional medical problems involving other organ systems), which accounts for 30% of genetic hearing loss cases, and non-syndromic (partial or entire loss of hearing that is not linked with other signs and symptoms) [4]. Non-syndromic hereditary hearing loss is characterized by significant genetic variability, with over 6000 causal variations discovered in over 110 genes to date [4]. Prelingual deafness is inherited in around 80% of cases, most of which are autosomal recessive and non-syndromic [5]. In many populations, the leading cause of severe-to-profound autosomal recessive non-syndromic hearing loss is a mutation in the GJB2 gene [6].

Advancements in next-generation sequencing (NGS) technologies have significantly transformed genetic testing and genome analysis methods, uncovering complex mutations associated with a variety of diseases. These sequencing techniques, both broad and targeted, are now pivotal in shaping research directions and therapeutic strategies. This progress has also broadened our understanding of the mutations in genes linked to hearing loss. Recognizing these genetic factors is essential for evaluating the progression of hearing loss and the extent to which natural hearing is preserved, which in turn informs subsequent treatment and rehabilitation efforts [7].

*CDH23* (cadherin-related 23) is a gene that is associated with various genetic disorders affecting hearing and vision. It is located on chromosome 10q21–q22 and it consists of 69 exons. In addition to that, it encodes cadherin 23, which is made up of 3354 amino acids and includes 27 extracellular domains (ECs), one transmembrane domain, and a short cytoplasmic domain [8,9]. Mutations in the *CDH23* gene can result in various conditions, including non-syndromic hearing loss (DFNB12), Usher syndrome type 1D, and age-related retinal degeneration [10]. In both the inner and outer hair cells of the cochlea, the *CDH23* gene is expressed and encodes glycoproteins that facilitate calcium-dependent cell–cell adhesion. The encoded *CDH23* protein plays a vital role in the structure of the tip link, helping to maintain the proper organization of stereocilia, the hair bundles in the inner ear, which are essential for hair cell function [9,11]. The *CDH23* protein is instrumental in establishing tip links through its interaction with protocadherin 15, encoded by the *PCDH15* gene (NM_001142771). This interaction is critical for preserving the structural integrity of tip links and supporting the mechanoelectrical transducer currents crucial for hair cell function [11]. Mutations in *CDH23* can significantly impair cadherin 23′s functionality. Such mutations are typically categorized as nonfunctional, commonly associated with Usher syndrome type 1D, or as hypofunctional missense mutations that cause non-syndromic hearing loss while maintaining retinal and vestibular functions, known as DFNB12 [12]. The presence of hypofunctional (DFNB12 allele) and nonfunctional (USH1D allele) mutations in trans configuration is thought to allow the partial function of the DFNB12 allele to preserve vision and vestibular abilities, predominantly affecting hair cells in the inner ear and leading to the DFNB12 hearing loss phenotype [12,13,14].

In this study, we investigated a consanguineous Emirati family affected by autosomal recessive non-syndromic hearing loss (ARNSHL) through clinical exome sequencing (CES). Our findings revealed a novel nonsense variant and a missense variant in the *CDH23* gene, highlighting the critical role of protein-coding genes in the complex auditory system and underscoring the significance of genetic analysis in understanding the etiology of ARNSHL.

## 2. Materials and Methods

### 2.1. Patients and Samples

Saliva samples were obtained from a consanguineous Emirati family affected by autosomal recessive non-syndromic hearing loss (ARNSHHL). Genomic DNA was isolated using the prep IT-L2P^®^ protocol from saliva collected with the Oragene-DNA Kit (OG-500) provided by DNA Genotek™, Stittsville, ON, Canada. Comprehensive audiological and medical assessments were performed, and informed consent was secured from all participants or their guardians. The University of Sharjah’s ethics committee approved all experimental protocols and consent procedures, ensuring adherence to established standards and regulations.

### 2.2. Clinical Exome Sequencing and Bioinformatic Analysis

Clinical exome sequencing (CES) was conducted on a *GJB2*-negative proband (II-1) using the Illumina HiSeq 2500 platform for next-generation sequencing (NGS). The DNA preparation, including end repair, addition, and adaptor ligation, utilized the SureSelect Clinical Research Exome V2 capture kit from Agilent Technologies, Santa Clara, CA, USA. The enriched DNA libraries were then sequenced on the same Illumina system. High-quality sequencing reads were aligned to the human reference genome (GRCh37/hg19) using the Burrows–Wheeler Aligner (BWA). Duplicate reads were managed using Picard tools, and variants were detected using the Genomic Analysis Toolkit (GATK). Variants were further annotated using the internal Variation and Mutation Annotation Toolkit (VariMAT v2.4.1), incorporating data from clinical-grade databases like GWAS and ClinVar, among others, and utilizing Ensembl and RefSeq for genomic annotation and functional analysis.

Variant filtering criteria included a Global Minor Allele Frequency (GMAF) below 0.001 or absence in databases such as ExAC Browser, 1000 Genomes, gnomAD, and dbSNP. Variants were also prioritized based on coding impact and their listing in genes associated with autosomal recessive non-syndromic deafness on the Hereditary Hearing Loss Homepage.

### 2.3. Sanger Sequencing

Initial screening of the *GJB2* gene was carried out by PCR amplification of its coding region using the Cx26-2F and Cx26-2R primers [15] as indicated in Table 1. The PCR amplicons were purified using ExoSAP-IT™ PCR Product Cleanup Reagent (Affymetrix, Fisher Scientific, Göteborg, Sweden) and then sequenced using the Big Dye Terminator V3.1 Cycle Sequencing Kit (Applied Biosystems, Waltham, MA, USA). Sequencing products underwent purification via EDTA/sodium acetate/ethanol precipitation and were analyzed with the 3500 Genetic Analyzer (Applied Biosystems, Thermo Fisher Scientific, Waltham, MA, USA). The sequences were aligned with the wild-type *GJB2* gene sequence (NM_004004.6) using the Basic Local Alignment Search Tool (BLAST). Additionally, to amplify exons 4 and 40 harboring the c.264G>A and c.5168G>A mutations in *CDH23*, specific primers listed in Table 1 were utilized. Amplified PCR products were similarly processed and sequenced. Capillary sequencing was performed on the Genetic Analyzer 3500 platform, and data analysis was conducted with Sequencing Analysis Software v6.0. The sequenced samples were compared to the *CDH23* NCBI Reference Sequence (NM_022124.6) using BLAST.

### 2.4. In Silico Analysis of c.264G>A and c.5168G>A Mutations

To further examine the effect of the mutations, the functions of the new variants c.264G>A and c.5168G>A were predicted and explored using SIFT [https://sift.bii.a-star.edu.sg/] (accessed on 15 August 2024), PolyPhen-2 [https://genetics.bwh.harvard.edu/pph2/] (accessed on 15 August 2024), and Mutation Taster [http://www.mutationtaster.org/] (accessed on 15 August 2024). Additionally, we employed the American College of Medical Genetics and Genomics (ACMG) criteria to assess and score the pathogenicity of these variants. This evaluation helped in classifying the variants according to established genetic standards, further detailed at [https://www.acmg.net/] (accessed on 28 October 2024).

### 2.5. Computational Methodologies for Predicting Mutation Impact

The Alphafold2 program was utilized to predict the three-dimensional structure of the wild-type *CDH23* protein and the mutated p. Arg1723His and p. Trp88Ter *CDH23* proteins.

The AlphaFold2 models of the *CDH23* protein with the mutations p.Trp88Ter and p.Arg1723His were determined using ColabFold (AlphaFold2 + MMSEQ2) [16,17,18]. We obtained the highest rank model and displayed it alongside the wild-type *CDH23* structure. The structures were determined using the following settings where the MSAs were obtained using the MMSEQ2 server, using three recycle steps with a greedy pairing strategy and template mode turned to none.

The AlphaFold2 models of the human *CDH23* wild-type 1-1400 and 1201-2001 residues were derived from the AlphaFold2 Protein Structure Database: AF_AFQ9H251F1 and were generated through ColabFold, respectively (alphafold.ebi.ac.uk) [16,17,18]. Structures were analyzed and displayed using the Open Source PyMOL Molecular Graphics (Version 2.6.0a0 Schrödinger, LLC, New York, NY, USA).

## 3. Results

### 3.1. Phenotype Characterization

**Figure 1:**
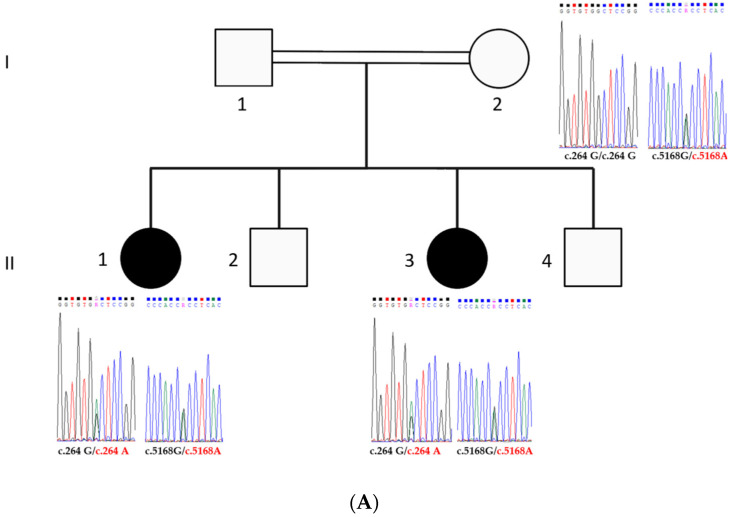

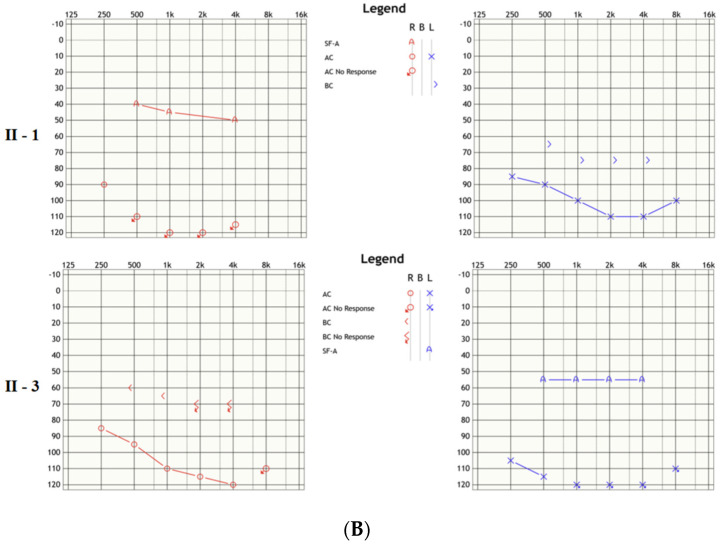
Pedigree, audiograms, and electropherograms. (**A**): Pedigree of the studied consanguineous family, with electropherograms illustrating the transmission of the c.264G>A and c.5168G>A mutations in the normal-hearing mother and the two affected daughters. (**B**): Audiograms showing hearing threshold tests for affected individuals II−1 and II−3. The audiograms present thresholds for the right ear (in red) and the left ear (in blue), both before and after cochlear implantation.

The study was conducted on a consanguineous Emirati family, featuring two deaf individuals (Figure 1A). Pedigree analysis indicated an autosomal recessive inheritance pattern, and clinical evaluations confirmed profound hearing loss without other anomalies in the siblings. Both daughters underwent cochlear implant surgeries, which improved their hearing from profound loss to moderate impairment, as illustrated in Figure 1B.

### 3.2. Clinical Exome Sequencing Data Analysis and In Silico Prediction Analysis

Mutations in the *GJB2* gene were screened to identify the disease-causing mutation in the investigated family using Sanger sequencing. The results indicated that the affected individuals tested negative for *GJB2* mutations. Consequently, clinical exome sequencing (CES) was performed only in the proband (II-1) to identify ARNSHL-causing variants in this family, generating 31,870 variants. After filtering out variants with a frequency of <0.001 in the gnomAD, 1000 Genomes, ExAC Browser, and dbSNP databases, the number of variants was reduced to 975. Further screening of rare DNA variants within genes associated with ARNSHL (https://hereditaryhearingloss.org/) (accessed on 13 January 2024) identified 12 potential variants (Table 2).

Our analysis identified two heterozygous variants in the *CDH23* gene (NM_022124.6) within the proband. The first is a previously documented guanine to adenine substitution (c.5168G>A, p.Arg1723His) and the second is a novel nonsense mutation (c.264G>A, p.Trp88Ter). To ascertain whether these variants are in trans or cis configuration, we conducted genetic analysis of the mother’s DNA as well as the DNA of the second affected daughter (II-3). Sanger sequencing of the mother confirmed that she carries only the c.5168G>A mutation in the heterozygous state, while she is homozygous normal for c.264G. This finding allows us to conclude that the affected daughters carry both mutations in trans, with the c.5168G>A mutation inherited maternally and the c.264G>A mutation inherited paternally. The c.5168G>A variant was predicted to be “probably damaging” and “disease-causing” by PolyPhen-2 and MutationTaster, respectively, but was considered “tolerated” by SIFT. According to ACMG criteria, this variant is classified as Likely Pathogenic due to the presence of PM1 and PM2 for its occurrence in a critical and well-established functional domain and its rarity in population databases, supported by PP3 for damaging predictions by computational tools. Meanwhile, the c.264G>A variant was predicted to be “disease-causing” by MutationTaster and is classified as Pathogenic under ACMG guidelines, with PVS1 due to its nonsense nature, PM2 for rarity, and supported by PP1 for segregation with the disease in multiple affected family members and PP3 for consistent pathogenic predictions.

### 3.3. Structural Alterations

**Figure 2:**
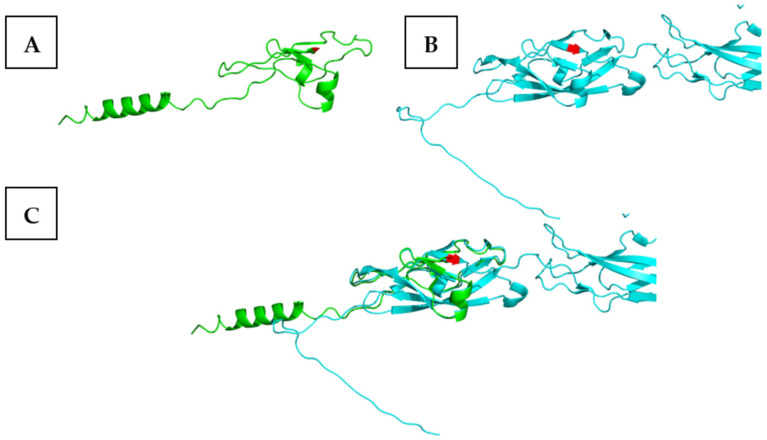
Representing the (**A**) p.Trp88Ter nonsense mutation, (**B**) wild-type, and (**C**) alignment of the p.Trp88Ter nonsense mutation with the wild-type model of the *CDH23* protein through the AlphaFold2 predicted model of the *CDH23* protein structure.

The structures of the p.Trp88Ter mutation result in a nonsense mutation with a nonfunctional short peptide fragment (Figure 2A). The wild-type structure (Figure 2B) was aligned to the wild-type structure resulting in an RMSD score of 0.710 (Figure 2C).

**Figure 3:**
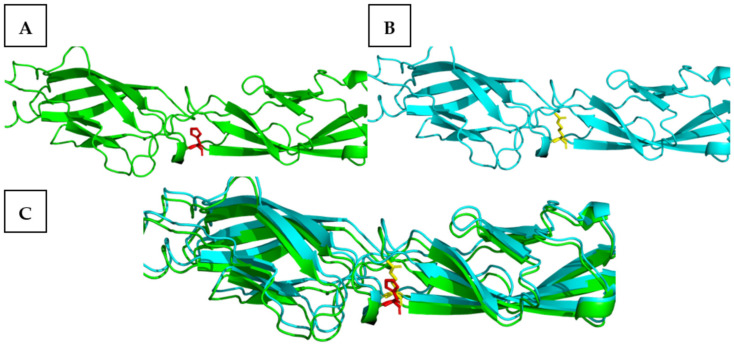
Representing (**A**) the p.Arg1723His missense mutation, (**B**) the wild-type sequence, and (**C**) the alignment of the p.Arg1723His missense mutation with the wild-type model of the AlphaFold2 predicted model of the *CDH23* protein structure.

The predicted model of the p.Arg1723His mutation (Figure 3A) was aligned to the wild-type *CDH23* (Figure 3B), which demonstrated a close alignment in structure (Figure 3C), resulting in an RMSD score of 1.149 from the predicted AlphaFold2 model of the wild-type *CDH23*, as observed in the figures below.

## 4. Discussion

Hearing loss (HL) is a prevalent sensory disorder affecting newborns globally, representing one of the most common sensory disorders. The *CDH23* gene, known for its association with hearing loss, harbors over 400 documented mutations that contribute significantly to non-syndromic hearing loss and are crucial for genetic screenings alongside genes like *GJB2* and *SLC26A4* [19]. Homozygosity mapping in consanguineous families has proven effective for identifying regions of homozygosity (ROH) and pinpointing genes implicated in recessive disorders. This approach highlights the increased likelihood of consanguineous spouses carrying identical recessive mutations, though compound heterozygosity is also possible and pertinent to these investigations [20].

In our study, we examined a consanguineous Emirati family affected by hereditary hearing loss, where both parents exhibited normal hearing and two daughters presented with severe-to-profound sensorineural hearing loss without any visual impairments. The utilization of clinical exome sequencing (CES) followed by Sanger sequencing validation facilitated the identification of compound heterozygous mutations in the *CDH23* gene, underscoring the efficacy of next-generation sequencing (NGS) technologies in diagnosing ARNSHL.

Our analysis highlighted two mutations in *CDH23*: the missense mutation c.5168G>A (p.Arg1723His) and the novel nonsense mutation c.264G>A (p.Trp88Ter). Previously, the p.Arg1723His variant had been identified in a heterozygous state in an individual with hearing loss and was found at low frequencies within the general population, as documented by the Exome Aggregation Consortium (ExAC, http://exac.broadinstitute.org (accessed on 13 January 2024); dbSNP rs189361642). Its low frequency does not preclude a pathogenic role, a position reinforced by our discovery of a second pathogenic variant, p.Trp88Ter. The presence of both mutations in trans in our study provides strong evidence supporting their combined pathogenic impact, further substantiated by the identification of a similar mutation, c.5168G>C (p.Arg1723Thr), in a Pakistani family with hearing loss [21]

The p.Trp88Ter mutation introduces a premature stop codon, likely resulting in nonsense-mediated decay or a truncated, nonfunctional protein, predicted as “disease-causing” by MutationTaster. This finding complements the deleterious impact of the p.Arg1723His mutation, emphasizing the necessity of comprehensive mutational analysis to fully understand the genetic interplay affecting hearing loss. Our study enriches the understanding of *CDH23′*s mutational landscape and illustrates the complex genetic architecture underlying hearing loss, highlighting the importance of detailed genetic scrutiny in elucidating the etiology of this common sensory disorder.

## 5. Conclusions

In conclusion, this study identified the association of *CDH23* nonsense and missense mutations—c.264G>A (p.Trp88Ter) and c.5168G>A (p.Arg1723His), respectively—with autosomal recessive non-syndromic hearing loss (ARNSHL) in a UAE family, leading to significant functional disruptions. Our results expand the mutation spectrum of *CDH23*, elucidate the phenotypic variability associated with *CDH23* mutant alleles, and establish a framework for understanding the genotypic hierarchy of *CDH23* mutations based on their pathogenic potential, as it is crucial in highly consanguineous populations, such as those in the UAE.

## Figures and Tables

**Table 1:** Primers used in this study.

Primer Name	Sequence (5′-3′)	
*GJB2-2F* *GJB2-2R* *CDH23-4F*	ACACGTTCAAGAGGGTTTGG GGGAAATGCTAGCGACTGAG GAGTGGTGGTTCCCTGATACCA	
*CDH23-4R*	AACCCTAAGTCCAGACACCAGC	
*CDH23-40F*	CAGTCTAGGGGAAACTGTGGGG	
*CDH23-40R*	TGACAGATGTAGCCACACAGGG	

**Table 2:** Candidate genes after filtration.

Gene	VARCLASS	CDNA_CHG	AA_CHG	Zygosity
*ADCY1* *WHRN* *MYO3A*	SILENT	c.2337C>G	p.Ala779=	Heterozygous
	MISSENSE	c.1091A>G	p.His364Arg	Heterozygous
	MISSENSE	c.2104T>G	p.Ser702Ala	Heterozygous
*CDH23* *CDH23*	NONSENSE	c.264G>A	p.Trp88Ter	Heterozygous
	MISSENSE	c.3619G>A	p.Val1207Met	Heterozygous
*CDH23* *OTOG* *TECTA* *TSPEAR*	MISSENSE	c.5168G>A	p.Arg1723His	Heterozygous
	SILENT	c.966G>A	p.Pro322=	Heterozygous
	MISSENSE	c.1738A>G	p.Ile580Val	Heterozygous
	INTRONIC	c.304-5652C>T	NA	Heterozygous
*TSPEAR*	INTRONIC	c.304-5665G>A	NA	Heterozygous
*TSPEAR* *TRIOBP*	INTRONIC	c.82+56914G>A	NA	Heterozygous
	SILENT	c.6630G>A	p.Ser2210=	Heterozygous

## Data Availability

All research data are available in the submitted article.

## References

[1] Lebeko K., Bosch J., Noubiap J.J.N., Dandara C., Wonkam A. (2015). Genetics of Hearing Loss in Africans: Use of Next Generation Sequencing is the Best Way Forward. Pan Afr. Med. J..

[2] Marazita M.L., Ploughman L.M., Rawlings B., Remington E., Arnos K.S., Nance W.E. (1993). Genetic Epidemiological Studies of Early—Onset Deafness in the US School—Age Population. Am. J. Med. Genet..

[3] Ohgami N., Iida M., Yajima I., Tamura H., Ohgami K., Kato M. (2013). Hearing Impairments Caused by Genetic and Environmental Factors. Environ. Health Prev. Med..

[4] Shearer A.E., Hildebrand M.S., Schaefer A.M., Smith R.J. (2023). Genetic Hearing Loss Overview. GeneReviews®[Internet]. https://www.ncbi.nlm.nih.gov/books/NBK1434/.

[5] Shearer A.E., Hildebrand M.S., Smith R.J. (2017). Hereditary Hearing Loss and Deafness Overview. https://www.ncbi.nlm.nih.gov/gtr/conditions/C0236038/.

[6] Sloan-Heggen C.M., Bierer A.O., Shearer A.E., Kolbe D.L., Nishimura C.J., Frees K.L., Ephraim S.S., Shibata S.B., Booth K.T., Campbell C.A. (2016). Comprehensive Genetic Testing in the Clinical Evaluation of 1119 Patients with Hearing Loss. Hum. Genet..

[7] Skarżyński H. (2021). The Role of Next Generation Sequencing in Predicting Hearing Loss. Expert Rev. Mol. Diagn..

[8] Chaïb H., Place C., Salem N., Dodé C., Chardenoux S., Weissenbach J., El Zir E., Loiselet J., Petit C. (1996). Mapping of DFNB12, a Gene for a Non-Syndromal Autosomal Recessive Deafness, to Chromosome 10q21–22. Hum. Mol. Genet..

[9] Usami S., Isaka Y., Miyagawa M., Nishio S. (2022). Variants in CDH23 Cause a Broad Spectrum of Hearing Loss: From Non-Syndromic to Syndromic Hearing Loss as Well as from Congenital to Age-Related Hearing Loss. Hum. Genet..

[10] Bork J.M., Peters L.M., Riazuddin S., Bernstein S.L., Ahmed Z.M., Ness S.L., Polomeno R., Ramesh A., Schloss M., Srisailpathy C.S. (2001). Usher Syndrome 1D and Nonsyndromic Autosomal Recessive Deafness DFNB12 are Caused by Allelic Mutations of the Novel Cadherin-Like Gene CDH23. Am. J. Hum. Genet..

[11] Kazmierczak P., Sakaguchi H., Tokita J., Wilson-Kubalek E.M., Milligan R.A., Müller U., Kachar B. (2007). Cadherin 23 and Protocadherin 15 Interact to Form Tip-Link Filaments in Sensory Hair Cells. Nature.

[12] Kim S.Y., Kim A.R., Kim N.K., Kim M.Y., Jeon E., Kim B.J., Han Y.E., Chang M.Y., Park W., Choi B.Y. (2015). Strong Founder Effect of P. P240L in CDH23 in Koreans and its Significant Contribution to Severe-to-Profound Nonsyndromic Hearing Loss in a Korean Pediatric Population. J. Transl. Med..

[13] Schultz J.M., Bhatti R., Madeo A.C., Turriff A., Muskett J.A., Zalewski C.K., King K.A., Ahmed Z.M., Riazuddin S., Ahmad N. (2011). Allelic Hierarchy of CDH23 Mutations Causing Non-Syndromic Deafness DFNB12 Or Usher Syndrome USH1D in Compound Heterozygotes. J. Med. Genet..

[14] Pennings R.J., Topsakal V., Astuto L., De Brouwer A.P., Wagenaar M., Huygen P.L., Kimberling W.J., Deutman A.F., Kremer H., Cremers C.W. (2004). Variable Clinical Features in Patients with CDH23 Mutations (USH1D-DFNB12). Otol. Neurotol..

[15] Tlili A., Al Mutery A., Kamal Eddine Ahmad Mohamed W., Mahfood M., Hadj Kacem H. (2017). Prevalence of GJB2 Mutations in Affected Individuals from United Arab Emirates with Autosomal Recessive Nonsyndromic Hearing Loss. Genet. Test. Mol. Biomark..

[16] Jumper J., Evans R., Pritzel A., Green T., Figurnov M., Ronneberger O., Tunyasuvunakool K., Bates R., Žídek A., Potapenko A. (2021). Highly Accurate Protein Structure Prediction with AlphaFold. Nature.

[17] Varadi M., Anyango S., Deshpande M., Nair S., Natassia C., Yordanova G., Yuan D., Stroe O., Wood G., Laydon A. (2022). AlphaFold Protein Structure Database: Massively Expanding the Structural Coverage of Protein-Sequence Space with High-Accuracy Models. Nucleic Acids Res..

[18] Mirdita M., Schütze K., Moriwaki Y., Heo L., Ovchinnikov S., Steinegger M. (2022). ColabFold: Making Protein Folding Accessible to All. Nat. Methods.

[19] Miyagawa M., Nishio S., Usami S. (2012). Prevalence and Clinical Features of Hearing Loss Patients with CDH23 Mutations: A Large Cohort Study. PLoS ONE.

[20] Ramzan K., Al-Numair N.S., Al-Ageel S., Elbaik L., Sakati N., Al-Hazzaa S.A., Al-Owain M., Imtiaz F. (2020). Identification of Novel CDH23 Variants Causing Moderate to Profound Progressive Nonsyndromic Hearing Loss. Genes.

[21] Shahzad M., Sivakumaran T.A., Qaiser T.A., Schultz J.M., Hussain Z., Flanagan M., Bhinder M.A., Kissell D., Greinwald Jr J.H., Khan S.N. (2013). Genetic Analysis through OtoSeq of Pakistani Families Segregating Prelingual Hearing Loss. Otolaryngol.-Head Neck Surg..

